# Calycosin inhibits porcine reproductive and respiratory syndrome virus replication and activates RIG-I/IRF3 signaling pathway

**DOI:** 10.3389/fvets.2025.1674259

**Published:** 2025-10-29

**Authors:** Yafei Chang, Zhaopeng Li, Mengqi Wang, Kanglei Pei, Xiaobo Chang, Jinyou Ma

**Affiliations:** ^1^College of Animal Science and Veterinary Medicine, Henan Institute of Science and Technology, Xinxiang, China; ^2^Postdoctoral Innovation Practice Base, College of Animal Science and Veterinary Medicine, Henan Institute of Science and Technology, Xinxiang, China

**Keywords:** PRRSV, calycosin, antiviral activity, RIG-I, IRF3

## Abstract

Porcine reproductive and respiratory syndrome virus (PRRSV), the causative pathogen of PRRS, remains one of the most important pathogens threatening the global pig industry. Due to the genetic diversity of PRRSV, the existing commercially vaccines cannot completely protect pigs from PRRSV infection. Flavonoid compounds play an important role in inhibiting viral replication. In this study, we found that calycosin, a natural active compound isolated from astragalus, could inhibit PRRSV replication regardless of whether calycosin was added pre-, co-, or post-PRRSV infection *in vitro*. Furthermore, coincubation of calycosin with PRRSV showed a better inhibitory effect compared to separate incubation, and calycosin mainly inhibited virus replication, assembly and release stages. Importantly, calycosin enhanced the antiviral immunity, as shown by the increased expression of IFN-β and ISG56. Subsequently, we discovered that calycosin promoted the expression of RIG-I and MAVS, and induced the phosphorylation and nuclear translocation of IRF3 during PRRSV infection. Collectively, calycosin could markedly inhibit PRRSV replication and activate the RIG-I/IRF3 signaling pathway. These data contribute to understanding the role of calycosin in PRRSV replication, and may provide valuable insights for the development of future antiviral strategies.

## Introduction

Porcine reproductive and respiratory syndrome virus (PRRSV) is a single-stranded positive-sense RNA virus that has caused enormous economic losses to the global swine industry (1–5). It is characterized by reproductive failure in sows and general respiratory symptoms in pigs of all ages and sexes (6). PRRSV is divided into two genotypes: PRRSV-1 and PRRSV-2 (7). According to phylogenetic tree analysis, PRRSV-1 can be classified into 4 lineages, whereas PRRSV-2 can be split into 11 lineages (8–10), so the prevalent forms of PRRSV are complex and variable. Currently, vaccination is the main means for preventing PRRS. However, the existing vaccines cannot completely protect pigs from PRRSV infection (11, 12). Thus, there is an urge to develop new efficacious therapies against PRRSV.

Innate immunity is the initial and crucial line of defense against virus infection. Following virus invasion, viral nucleic acids, identified as pathogen-associated molecular patterns (PAMPs), are recognized by pattern-recognition receptors (PRRs), thereby activating associated signaling pathways (13–15). Retinoic acid-inducible gene I (RIG-I)-like receptors (RLRs), as the pivotal RNA sensor, could recruit the mitochondrial antiviral signaling (MAVS) and subsequently trigger downstream signals to resist viral infection (16–18).

Calycosin, a natural isoflavone primarily found in Astragalus membranaceus, plays a pivotal role as a regulator in the context of multiple diseases and biological processes, such as cell apoptosis, anti-tumor and anti-inflammatory (19). Previous studies have reported that calycosin exerts anti-HCC activity by targeting TRX1 to regulate oxidative stress and promote mitochondria-mediated apoptosis (20). In another report, yang et al. demonstrated that calycosin can effectively downregulate the expression of HMGB1 and TLR4. Additionally, it decreases the phosphorylation of NF-κB and IκB, and reduces the secretion of inflammatory cytokines such as IL-6 and IL-18 (21). More recently, calycosin has been demonstrated to be able to inhibit the lytic replication of KSHV and suppress inflammatory cytokines such as IL-6 and IL-8 induced by KSHV infection (22). Nevertheless, whether calycosin has antiviral effects on PRRSV remains unknown.

In this study, we evaluated the antiviral activity of calycosin on PRRSV replication in Marc-145 cells and found that calycosin could suppress the replication, assembly and release stages of PRRSV infection. Remarkably, calycosin activated the RIG-I/IRF3-mediated I-IFN signaling pathway. Overall, this study highlighted the role of calycosin in the replication of PRRSV for the first time, and our findings may provide insights into a potential therapeutic approach for PRRSV control.

## Materials and methods

### Cells and virus

Marc-145 cells (the PRRSV permissive cell line) were maintained in Dulbecco’s modified Eagle’s medium (DMEM, Solarbio) supplemented with 10% fetal bovine serum (FBS, Gibco), 100 U/mL penicillin (Solarbio) and 100 μg/mL streptomycin (Solarbio) at 37 °C and under 5% CO_2_. PRRSV strain BJ-4 (GenBank No. AF331831) stored in our laboratory was a kind gift from Prof. Hanchun Yang (China Agricultural University).

### Antibodies and reagents

The RIG-I antibody (20566-1-AP), MAVS antibody (14341-1-AP), p-IRF3 antibody (29528-1-AP), IRF3 antibody (11312-1-AP), p-STAT1 antibody (28979-1-AP), STAT1 antibody (66545-1-Ig), Beta Actin antibody (66009-1-Ig), GAPDH antibody (60004-1-Ig), Histone H3 antibody (17168-1-AP), HRP-conjugated goat anti-rabbit IgG (SA00001-2) and HRP-conjugated goat anti-mouse IgG (SA00001-1) were purchased from Proteintech. The anti-PRRSV N protein antibody (GTX129270) was purchased from GeneTex. Goat anti-rabbit IgG-FITC antibody (F0382-.5ML) was purchased from Sigma-Aldrich. All primary and secondary antibodies used for western blotting were diluted according to the instructions provided with the antibody reagents. Quercetin, isoquercitrin, taxifolin, astilbin, calycosin, apigenin, puerarin, and ipriflavone were purchased from Solarbio. The compounds mentioned in the text need to be dissolved in DMSO and the final concentration of DMSO in the working solution is kept below 0.1%.

### Cytotoxicity assay

TransDetect® cell counting kit (CCK) (TransGen Biotech) was used to determine the viability of Marc-145 cells. In brief, the cells were incubated with the corresponding drugs for 48 h at 37 °C, then 100 μL of 10% CCK solution was added according to the manufacturer’s protocol. After 1 h of incubation, absorbance was measured at 450 nm with a microplate reader.

### Quantitative real-time PCR analysis

The RNA transcription levels of PRRSV ORF7 in Marc-145 cells from different groups were determined by quantitative real-time PCR (qPCR). The total RNAs were extracted using Tissue/Cell Total RNA Isolation Kit (RC113-01, Vazyme), and reverse transcription was performed with HiScript® III All-in-one RT SuperMix Perfect for qPCR (R333-01, Vazyme). The cDNA products were amplified using ChamQ Universal SYBR qPCR Master Mix (Q711-02, Vazyme). The reaction conditions for qPCR were set in accordance with the product instructions, and the real-time quantitative PCR instrument was the QuantStudio™ 5 from ThermoFisher. The relative mRNA levels were calibrated to the level of GAPDH and calculated using the 2^−ΔΔCT^ method. The primers for qPCR are listed in Table 1.

**Table 1:** Primers used for qPCR.

Primers	Forward primer (5′–3′)	Reverse primer (5′–3′)
ORF7	AAACCAGTCCAGAGGCAAGG	GCAAACTAAACTCCACAGTGTAA
IFN-β	ACGGCTCTTTCCATGAGCTAC	GTCAATGCAGCGTCCTCCTT
ISG56	AGGAAACACCCACTTCGGTC	CCTCTAGGCTGCCCTTTTGT
GAPDH	GAAGGTGAAGGTCGGAGTCA	CATGTAAACCATGTAGTTGAGGTC

### Western blot

The cells were washed three times with cold PBS, and then lysed with RIPA lysis buffer (Sigma-Aldrich) supplemented with protease inhibitor cocktail (Roche) and PhosSTOP phosphatase inhibitor (Roche). When indicated, nuclear and cytoplasmic proteins were separated by nuclear and cytoplasmic protein extraction kit (Beyotime). Then, the protein samples were subjected to SDS-PAGE and transferred onto a 0.22 μm PVDF membrane (Millipore). The membranes were blocked with 5% skim milk (BD Biosciences) for 2 h at room temperature. After washing with PBST, membranes were incubated overnight at 4 °C with indicated primary antibody, followed by the incubation with appropriate HRP-conjugated secondary antibodies for 1 h at room temperature. After washing with PBST, the distinct protein bands were detected by ECL western blot substrate (Thermo Fisher Scientific).

### Immunofluorescence assay (IFA)

Marc-145 cells in 24-well plates were fixed with 4% paraformaldehyde (Beyotime) for 20 min, permeabilized with 0.2% Triton X-100 for 10 min and blocked with 5% skim milk for 1 h at room temperature. Then the primary antibody (anti-PRRSV N Ab) was added and incubated overnight at 4 °C. Subsequently, Goat anti rabbit IgG FITC antibody was added at room temperature for 1 h. Finally, the cells were stained with DAPI (Beyotime) for 10 min, and the immunofluorescence was observed using a Zeiss inverted fluorescence microscope.

### Time-of-addition assay

Marc-145 cells were seeded in 6-well plates at 37 °C, following the mock treatment (untreated: no calycosin was added), post-treatment (calycosin (40 μg/mL) was added 2 h after PRRSV infection), co-treatment (calycosin (40 μg/mL) and PRRSV were added together), or pre-treatment (calycosin (40 μg/mL) was added 2 h before PRRSV infection). After washing, the samples were collected at 24 hpi and subjected to indicated assays. The final concentration of DMSO in the working solution was maintained at 0.04%.

**Figure 1:**
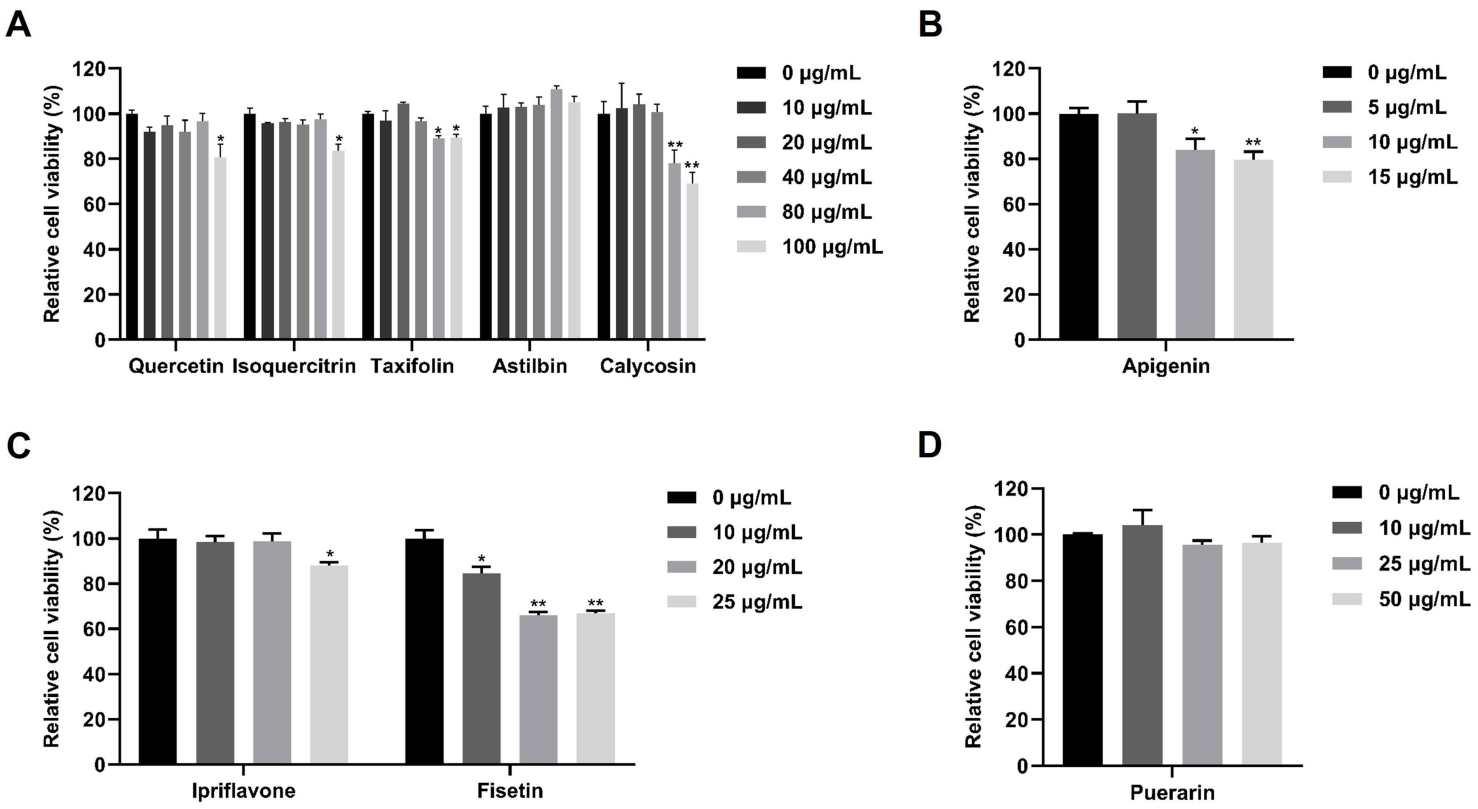
Cell viability of flavonoids in Marc-145 cells. Marc-145 cells were treated with nine different flavonoids at the indicated concentrations. After 24 h, the cell viability was determined by CCK-8 assay. **(A)** The cell viability of quercetin, isoquercitrin, taxifolin, astilbin and calycosin. **(B)** The cell viability of apigenin. **(C)** The cell viability of ipriflavone and fisetin. **(D)** The cell viability of puerarin. The asterisks in the figures indicate significant differences (*p < 0.05, **p < 0.01).

### Viral binding, entry, replication, assembly, and release assays

Virus binding, entry, replication, assembly, and release assays were performed according to published reports (23–25).

Virus binding assay. Marc-145 cells were pre-cooled at 4 °C for 2 h, and then were treated simultaneously with PRRSV at a multiplicity of infection (MOI) of 5 and calycosin (40 μg/mL) at 4 °C for another 2 h. Next, the cells were washed with ice-cold PBS three times, and samples were collected to measure the viral RNA.

Virus entry assay. Marc-145 cells were cultured at 4 °C for 2 h before PRRSV infection (MOI = 5) for 2 h at 4 °C. After washing, the cells were incubated with calycosin (40 μg/mL) or DMSO at 37 °C for 2 h. Then the samples were harvested and quantified by qPCR.

Virus replication assay. Marc-145 cells were infected with PRRSV (MOI = 0.1) at 37 °C for 6 h. After washing three times, the cells were treated with calycosin (40 μg/mL) or DMSO at 37 °C. At 24 h post-infection (hpi), the samples were collected for the corresponding assay.

Virus assembly assay. Marc-145 cells were incubated with the mixture of PRRSV and calycosin (40 μg/mL) at 37 °C for 2 h. The cells were washed with PBS three times and subsequently treated with calycosin for another 22 h. At 24 hpi, the cells and supernatants were collected for the measurement of viral titers and viral RNA.

Virus release assay. Marc-145 cells were infected with PRRSV (MOI = 0.1), and at 24 hpi, the cells were washed three times and then treated with calycosin (40 μg/mL) or DMSO for 10, 30, and 60 min at 37 °C. Next, the cell supernatants were harvested for the measurement of viral titers.

### Virus titration

Virus titers were determined according to a previous report (26). Briefly, Marc-145 cells grown in 96-well plates were infected with 10-fold serial dilutions of samples. After 2 h of incubation, the supernatants were replaced with DMEM supplemented with 2% FBS, and the viral titers were calculated at 4 days post-infection (dpi) and expressed as log_10_TCID_50_/mL.

### Statistical analysis

All of the data were presented as group mean and standard deviation (SD) and analyzed by Student t-test. All experiments were repeated at least three times. An unadjusted *p* value of < 0.05 was considered statistically significant.

## Results

### Calycosin exhibits anti-PRRSV activity

**Figure 2:**
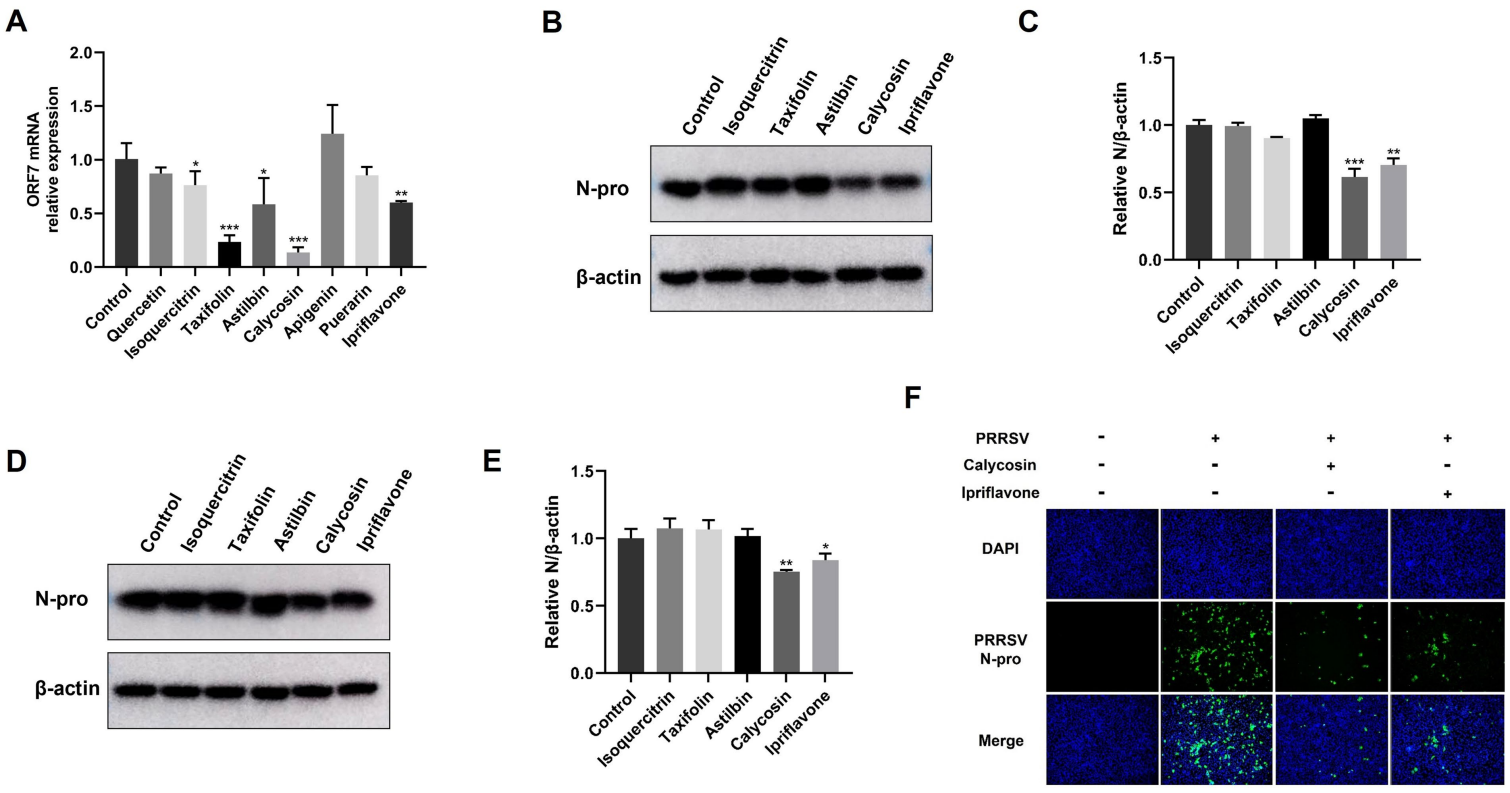
Identification of anti-PRRSV activity of flavonoids in Marc-145 cells. **(A)** Marc-145 cells were infected with PRRSV at a MOI of 0.1 and then simultaneously treated with quercetin (80 μg/mL), isoquercitrin (80 μg/mL), taxifolin (40 μg/mL), astilbin (100 μg/mL), calycosin (40 μg/mL), apigenin (5 μg/mL), puerarin (50 μg/mL) and ipriflavone (20 μg/mL). After 24 h, the cells were collected, and the mRNA levels of PRRSV ORF7 were assessed using qPCR. **(B,D)** Marc-145 cells were infected with PRRSV at a MOI of 0.1, followed by respective treatment with isoquercitrin (80 μg/mL), taxifolin (40 μg/mL), astilbin (100 μg/mL), calycosin (40 μg/mL) and ipriflavone (20 μg/mL). After 24 and 48 h, the PRRSV N protein levels were detected by western blotting. **(C,E)** The relative expression levels of N protein were quantified using Image J software at 24 and 48 hpi. **(F)** Marc-145 cells were infected with PRRSV at a MOI of 0.1 and then treated with calycosin (40 μg/mL) and ipriflavone (20 μg/mL), respectively. At 24 hpi, the cells were fixed and the expression of PRRSV N was analyzed by IFA. The asterisks in the figures indicate significant differences (*p < 0.05, **p < 0.01, ***p < 0.001).

To investigate the potential anti-PRRSV effects of flavonoids *in vitro*, we selected a range of compounds, including quercetin, isoquercitrin, taxifolin, astilbin, calycosin, apigenin, puerarin, and ipriflavone. Cell viability of these flavonoids on Marc-145 cells was tested using the CCK8 assay. As shown in Figure 1, when the maximum concentrations tested were determined to be 80 μg/mL for quercetin and isoquercitrin, 40 μg/mL for taxifolin and calycosin, 100 μg/mL for astilbin, 5 μg/mL for apigenin, 20 μg/mL for ipriflavone, and 50 μg/mL for puerarin, the cell viability was not significantly impaired. Subsequently, the anti-PRRSV effects of flavonoids were assessed by detecting the expression levels of PRRSV ORF7 and N protein in Marc-145 cells. The results showed that isoquercitrin, taxifolin, astilbin, calycosin, and ipriflavone significantly decreased the mRNA levels of PRRSV ORF7 compared to the control group (Figure 2A). However, western blot analysis showed that only calycosin and ipriflavone markedly decreased the expression levels of N protein at 24 and 48 hpi (Figures 2B–E). Likewise, the reduction of PRRSV infection in Marc-145 cells treated with calycosin or ipriflavone was visualized by the IFA (Figure 2F). Overall, calycosin and ipriflavone exhibited the most significant anti-PRRSV effect, and calycosin was used for a more in-depth study.

**Figure 3:**
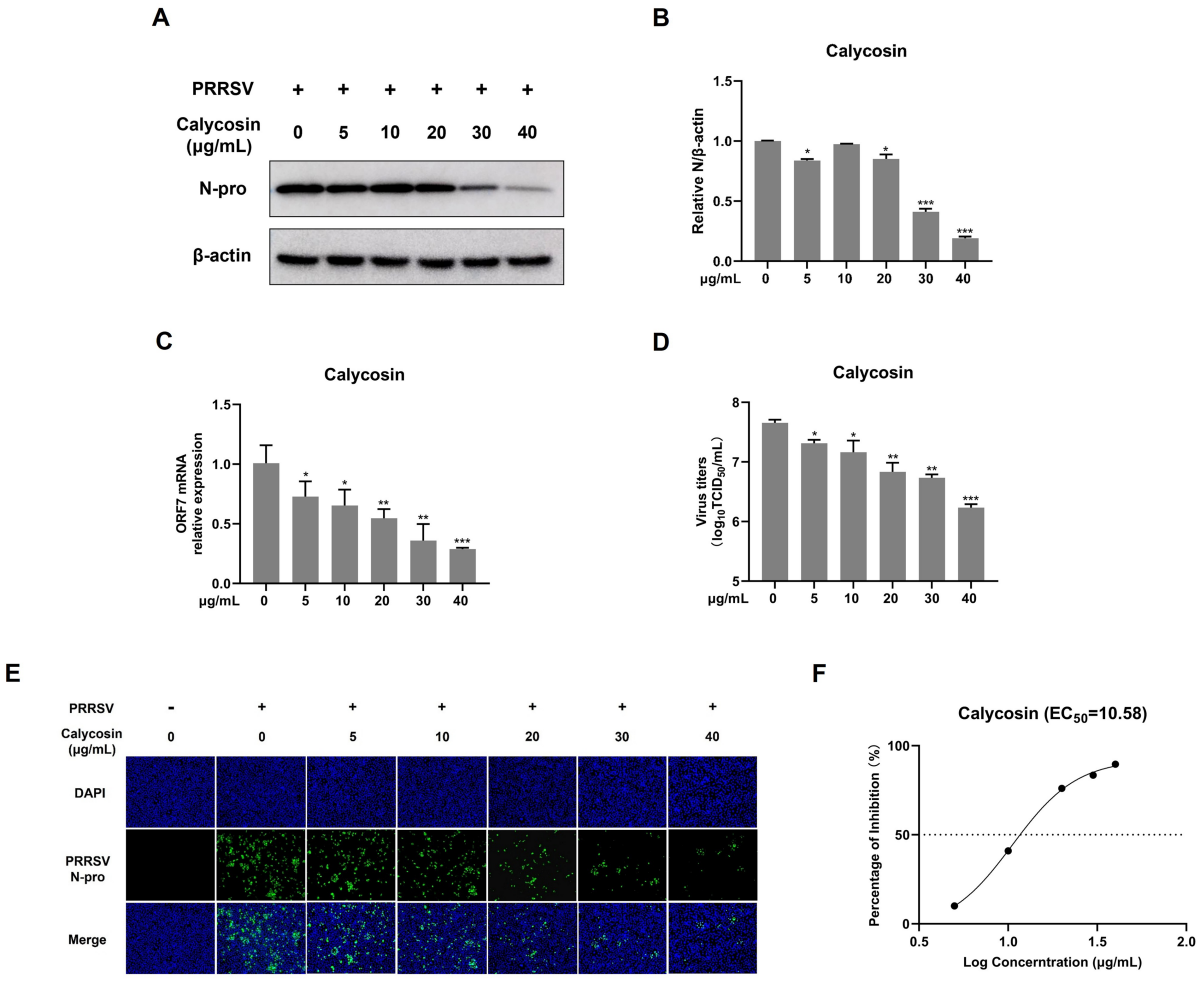
Calycosin inhibits PRRSV replication in a dose-dependent manner. Marc-145 cells were infected with PRRSV (MOI = 0.1) in the presence of calycosin (0, 5, 10, 20, 30, 40 μg/mL) for 24 h. **(A)** The expression levels of PRRSV N protein were detected by western blotting. **(B)** The relative expression levels of N protein were quantified using Image J software. **(C)** The mRNA levels of PRRSV ORF7 were examined by qPCR. **(D)** The virus titers were analyzed by TCID_50_. **(E)** The expression levels of PRRSV N protein were detected by IFA. **(F)** EC_50_ of calycosin on PRRSV. The asterisks in the figures indicate significant differences (*p < 0.05, **p < 0.01, ***p < 0.001).

### Calycosin inhibits PRRSV replication in a dose-dependent manner

To conduct a more systematic investigation into the effects of calycosin on PRRSV, we first examined the impact of different concentrations (ranging from 0 to 40 μg/mL) of calycosin on PRRSV using western blot, qPCR, viral titers, and IFA. As shown in Figures 3A,B, calycosin significantly reduced the abundance of PRRSV N protein at 20, 30, and 40 μg/mL compared to the control group, and calycosin showed the strongest inhibitory effect at a concentration of 40 μg/mL. Meanwhile, calycosin decreased the mRNA levels of PRRSV ORF7 (Figure 3C), and the viral titers in the supernatants of calycosin-treated cells were lower than those in corresponding control cells at different concentrations (Figure 3D), which was consistent with the IFA results (Figure 3E). In addition, the EC_50_ of calycosin was 10.58 μg/mL (Figure 3F). Together, these results indicated that calycosin suppressed PRRSV replication in a dose-dependent manner.

**Figure 4:**
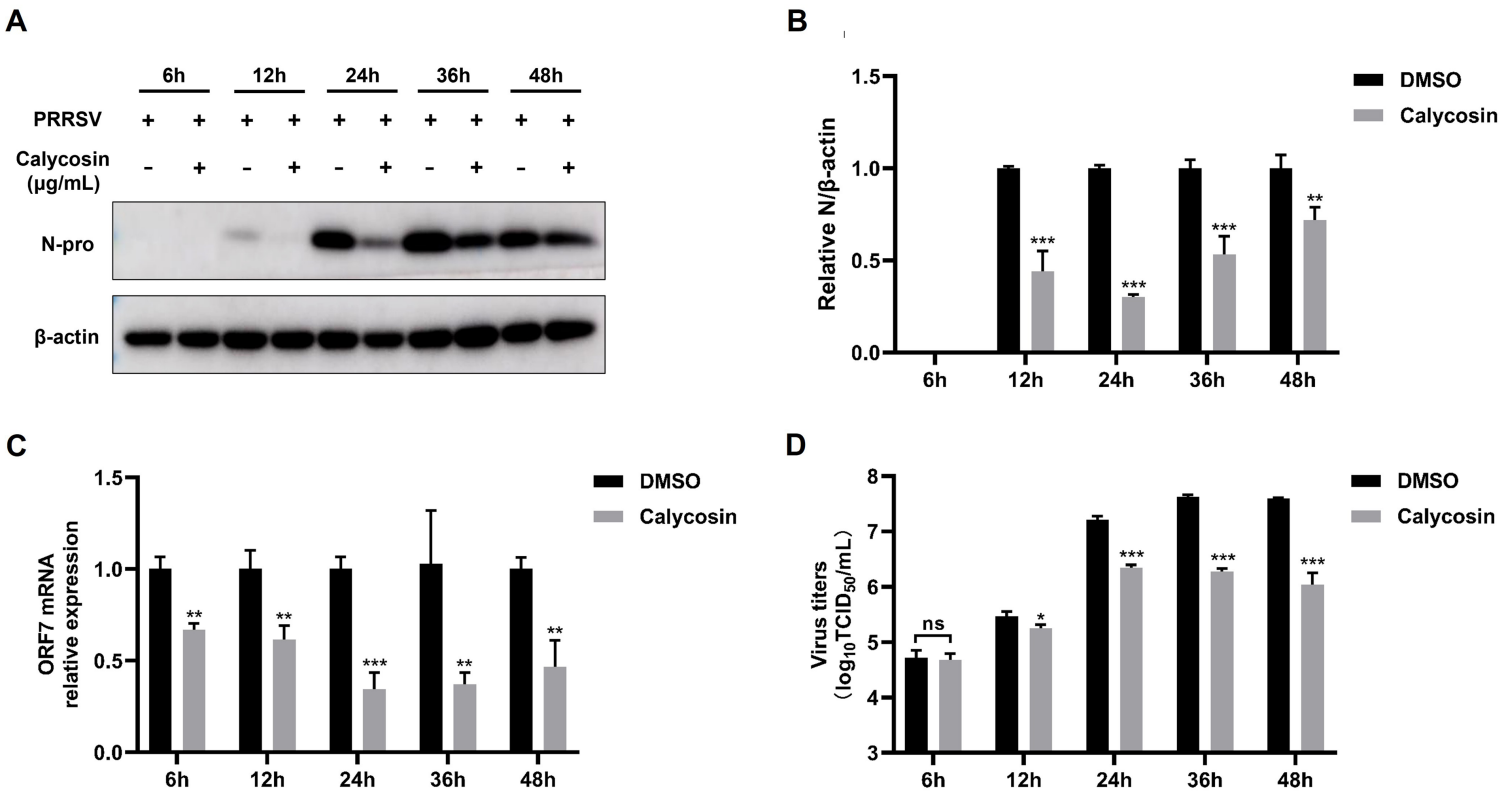
Calycosin inhibits PRRSV replication at different time points. Marc-145 cells were infected with PRRSV (MOI = 0.1) in the absence or presence of calycosin (40 μg/mL). At 6, 12, 24, 36, and 48 hpi, the cells were collected. **(A)** The expression levels of PRRSV N protein were detected by western blotting. **(B)** The relative expression levels of N protein were quantified using Image J software. **(C)** The mRNA levels of PRRSV ORF7 were examined by qPCR. **(D)** The virus titers were analyzed by TCID_50_. The asterisks in the figures indicate significant differences (*p < 0.05, **p < 0.01, ***p < 0.001, and ns, no significance).

In addition, we also investigated the effects of calycosin on PRRSV at different time points. Western blot confirmed that the expression of the PRRSV N protein was decreased relative to cells untreated with calycosin at 12, 24, 36, and 48 h post-infection (hpi) (Figures 4A,B). Furthermore, the results of qPCR and TCID_50_ assays also indicated that calycosin significantly inhibited PRRSV infection at different time points (Figures 4C,D).

**Figure 5:**
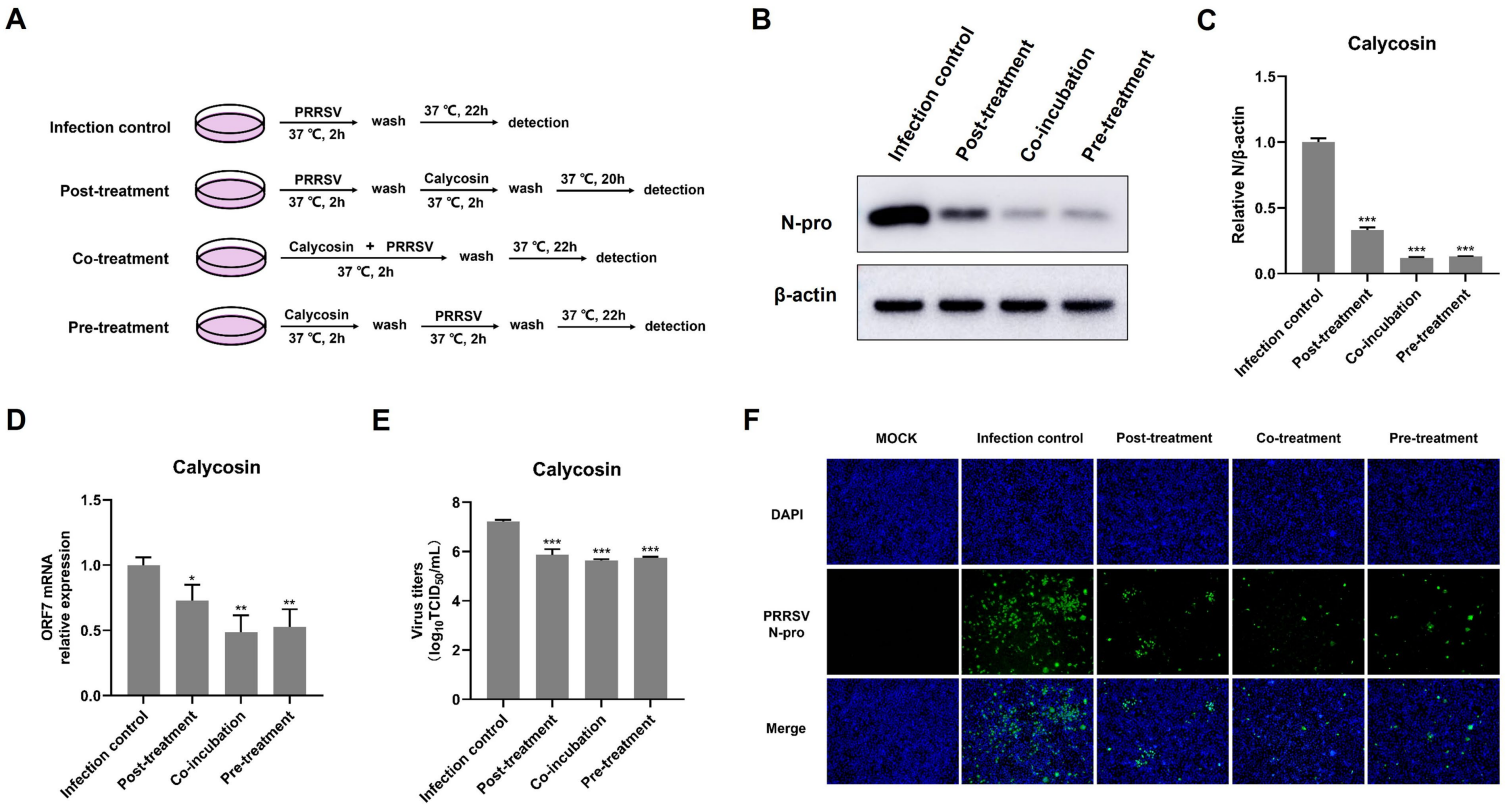
Calycosin suppresses PRRSV replication in various treatment stages in the time-of-addition assay. **(A)** Schematic diagram of experimental setup of calycosin (40 μg/mL) and PRRSV (MOI = 0.1) treatments in Marc-145 cells. **(B,F)** PRRSV N protein levels were detected by western blotting and IFA. **(C)** The relative expression levels of N protein were quantified using Image J software. **(D)** The mRNA levels of PRRSV ORF7 were examined by qPCR. **(E)** The virus titers were analyzed by TCID_50_. The asterisks in the figures indicate significant differences (*p < 0.05, **p < 0.01, ***p < 0.001).

### Calycosin disturbs PRRSV infection at different treatment stages

To further explore the anti-PRRSV mechanisms of calycosin *in vitro*, we examined the anti-PRRSV effects of calycosin under different treatment conditions as designed in Figure 5A. The results showed that calycosin significantly reduced the abundance of the PRRSV N protein, the mRNA levels of PRRSV ORF7, and the viral titers at all treatment stages (Figures 5B–E). In particular, co-treatment with calycosin exhibited the strongest inhibitory effect. The inhibitory effect on PRRSV following post-treatment with calycosin was slightly less than that observed with co- or pre-treatment with calycosin. Similarly, the reduction of PRRSV infection in cells treated with calycosin in different ways was visualized by IFA (Figure 5F). These results suggested that calycosin had the capacity to inhibit PRRSV infection and might act in both direct and indirect manners.

### Calycosin reduces the infectivity of PRRSV

**Figure 6:**
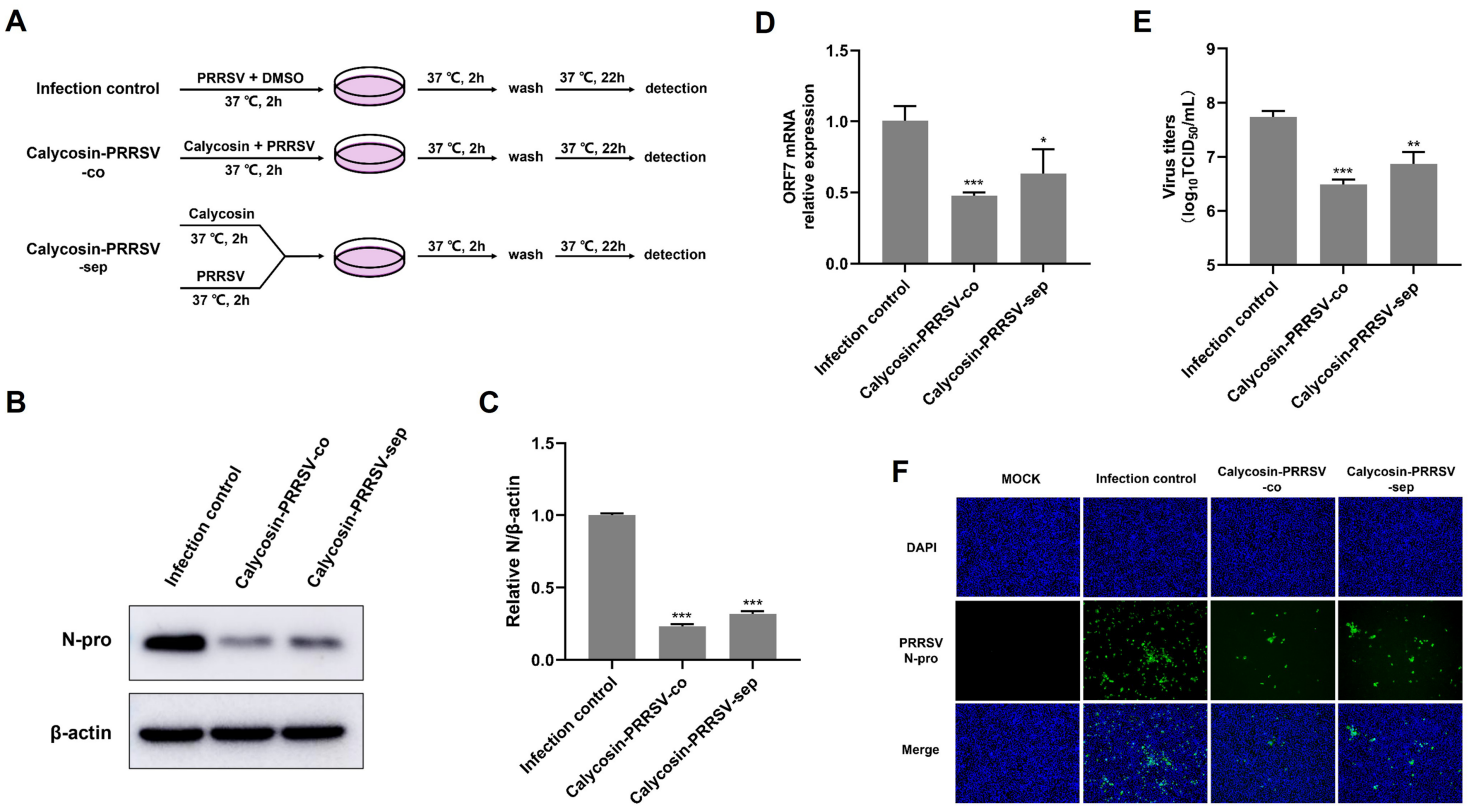
Calycosin directly reduces the infectivity of PRRSV. **(A)** Schematic of experimental setup of calycosin (40 μg/mL) and PRRSV (MOI = 0.1) treatments in Marc-145 cells. **(B,F)** PRRSV N protein levels were detected by western blotting and IFA. **(C)** The relative expression levels of N protein were quantified using Image J software. **(D)** The mRNA levels of PRRSV ORF7 were examined by qPCR. **(E)** The virus titers were analyzed by TCID_50_. The asterisks in the figures indicate significant differences (*p < 0.05, **p < 0.01, ***p < 0.001).

In order to conduct an in-depth study on whether calycosin has a direct inhibitory effect on PRRSV, Marc-145 cells were treated with PRRSV-DMSO-co, PRRSV-calycosin-co, and PRRSV-calycosin-sep as shown in Figure 6A, and then the results were determined using western blot, qPCR, TCID_50_ and IFA. The results indicated that co-incubation of calycosin and PRRSV for 2 h (PRRSV-calycosin-co) significantly reduced the levels of N protein (Figures 6B,C), PRRSV ORF7 mRNA (Figure 6D), and virus titers (Figure 6E) in the virus-infected cells compared to the PRRSV-DMSO-co group and the PRRSV-calycosin-sep group. These findings were consistent with the IFA results (Figure 6F). Overall, these results suggest that co-incubation with calycosin can reduce the infectivity of PRRSV.

### Calycosin blocks replication, assembly, and release of PRRSV

**Figure 7:**
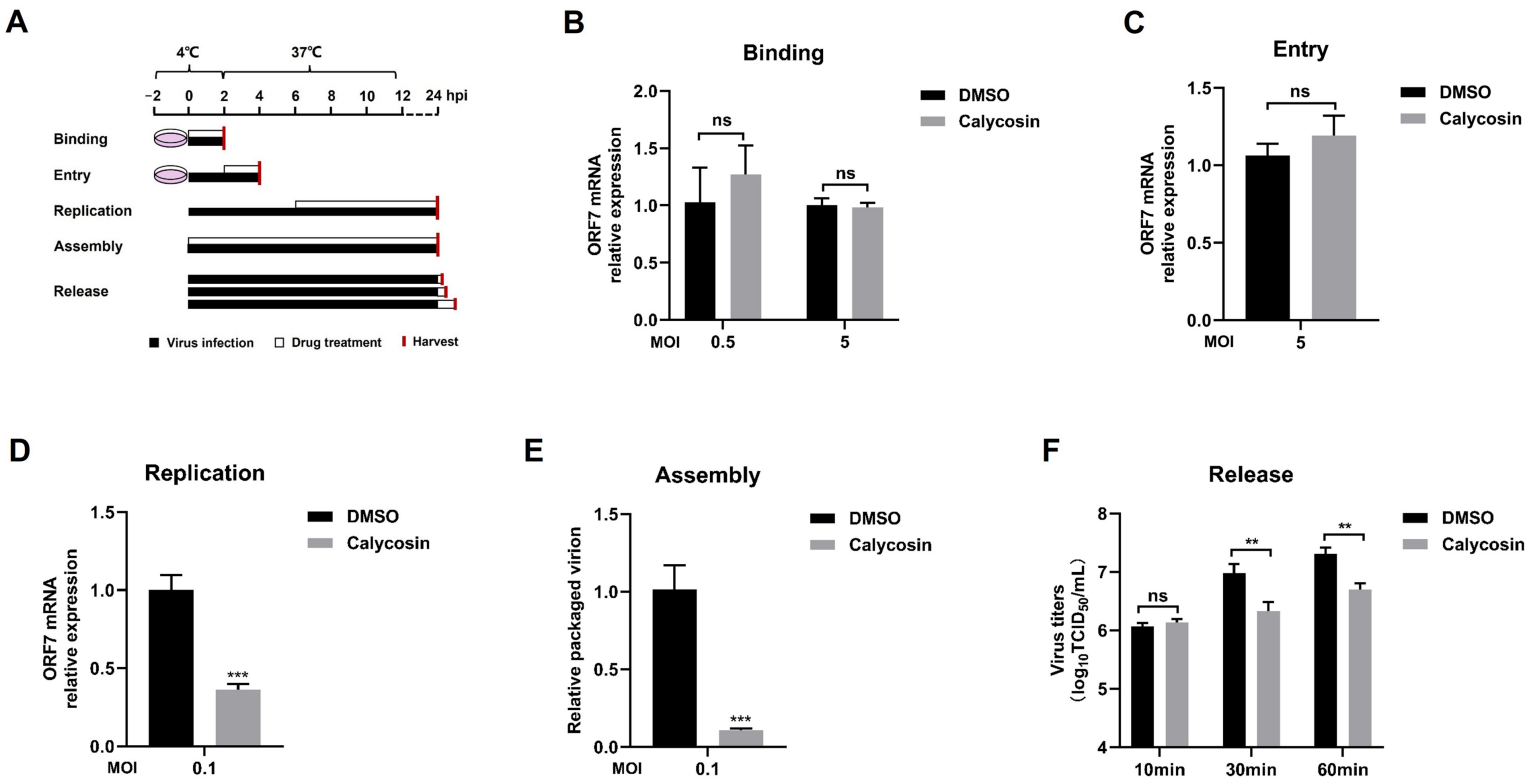
Calycosin blocks the replication, assembly, and release of the PRRSV but not the entry and binding stage. **(A)** Schematics of viral binding, entry, replication, assembly, and release assay. **(B–D)** The PRRSV ORF7 mRNA levels were determined by qPCR in the virus binding, entry, and replication assay. GAPDH was used as reference control. **(E)** The relative packaging efficiency of the viral genome was indicated by the ratio of viral titers to the copy number of the total viral genome. **(F)** In the virus release assay, virus titration was carried out through TCID_50_ calculation based on cell supernatant. The asterisks in the figures indicate significant differences (*p < 0.05, **p < 0.01, ***p < 0.001, and ns, no significance).

Next, we investigated whether calycosin inhibited the binding, entry, replication, assembly, and release stages of PRRSV infection. Marc-145 cells were infected with PRRSV, and calycosin treatment was applied at different stages as shown in Figure 7A. The results showed that no significant difference was observed in the binding and entry experiments between untreated and calycosin-treated group (Figures 7B,C). However, calycosin could reduce the mRNA levels of PRRSV ORF7 during virus replication stage (Figure 7D). The ratio of the packaged genome was significantly reduced by calycosin treatment, which suggests calycosin exhibits an inhibitory effect on PRRSV assembly (Figure 7E). In addition, calycosin could also affect PRRSV infection during the virus release stage (Figure 7F). These results indicated that calycosin blocked virus replication, assembly, and release stages.

### Calycosin markedly activates RIG-I/IRF3 signaling pathway

**Figure 8:**
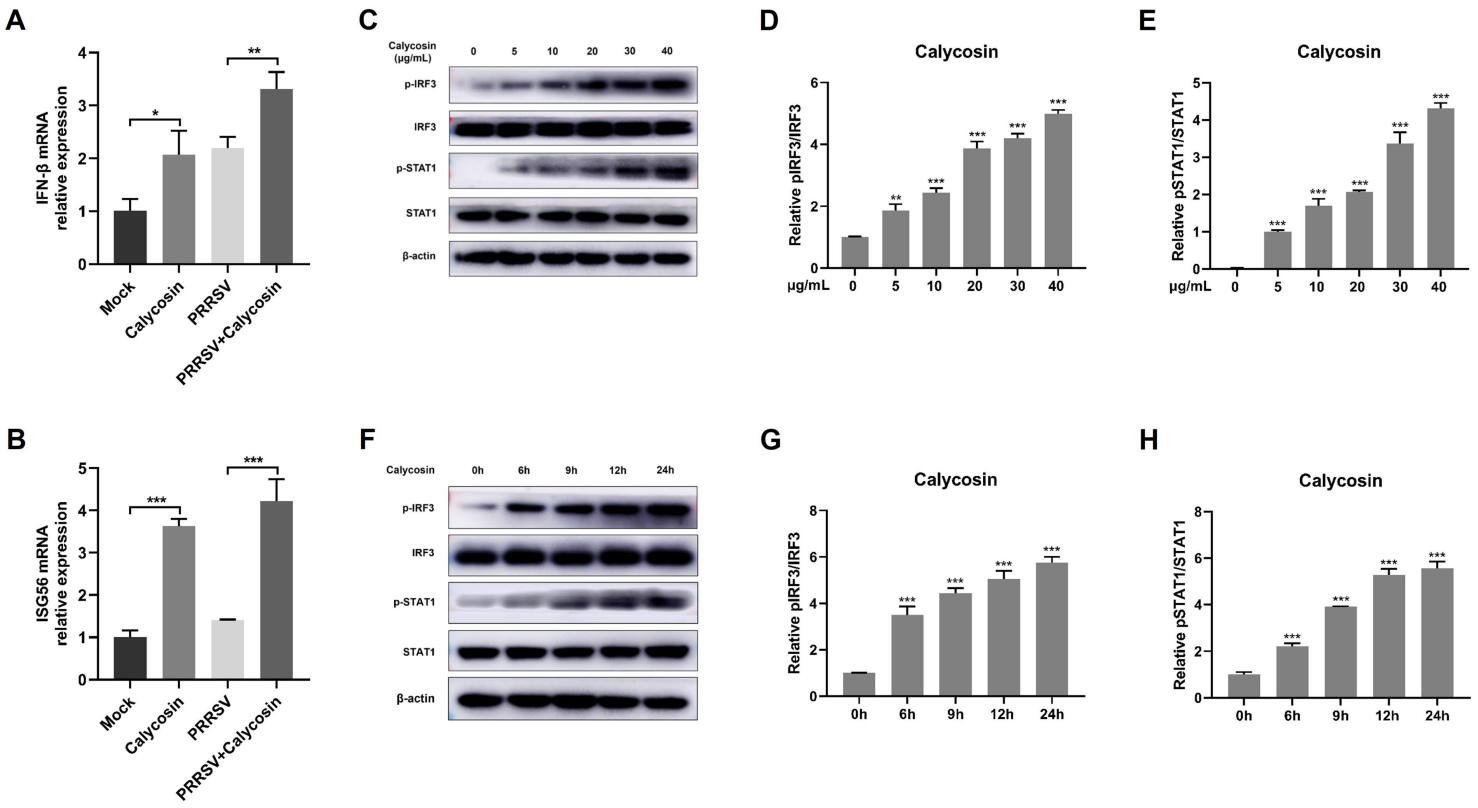
Calycosin effectively induced the activation of I-IFN signaling. Marc-145 cells were incubated with calycosin (40 μg/mL) and infected with or without PRRSV (MOI = 0.1) for 24 h, then the mRNA levels of IFN-β **(A)** and ISG56 **(B)** were measured by qPCR. Marc-145 cells were treated with calycosin (0, 5, 10, 20, 30, 40 μg/mL) for 24 h. **(C)** The pIRF3, IRF3, pSTAT1, and STAT1 were detected by western blotting. β-actin was used as an internal control. **(D,E)** The relative expression levels of pIRF3 and pSTAT1 were quantified using Image J software. **(F–H)** The levels of pIRF3, IRF3, pSTAT1, and STAT1 were also detected in cells treated with calycosin (40 μg/mL) at different times (0, 6, 9, 12, and 24 h) by western blotting. The asterisks in the figures indicate significant differences (*p < 0.05, **p < 0.01, ***p < 0.001).

Type I-IFN signaling plays a crucial role in restricting virus infection (13, 27). We next determined whether calycosin regulates the production of IFN-β signaling during PRRSV infection. Herein we found that calycosin could induce IFN-β mRNA transcription in the absence of virus and further enhance the levels of IFN-β induced by PRRSV (Figure 8A). Additionally, the transcription levels of ISG56 were also upregulated by calycosin during PRRSV infection (Figure 8B).

Subsequently, we investigated the underlying mechanisms of calycosin in the innate immune response to PRRSV. As we know, RIG-I-like receptors (RLRs) and their adaptor protein MAVS are key components of the host machinery for RNA virus recognition and play a crucial role in triggering the antiviral innate immune response against RNA viruses (18, 28–30). To investigate the potential impact of calycosin treatment on RIG-I signaling pathway, we first investigated the effect of calycosin on the activation of IRF3 under different concentrations and durations. Herein, we found that calycosin could induce phosphorylation of IRF3 and STAT1. Moreover, the degree of phosphorylation exhibited a marked increase in a dose- and time-dependent manner (Figures 8C–H).

**Figure 9:**
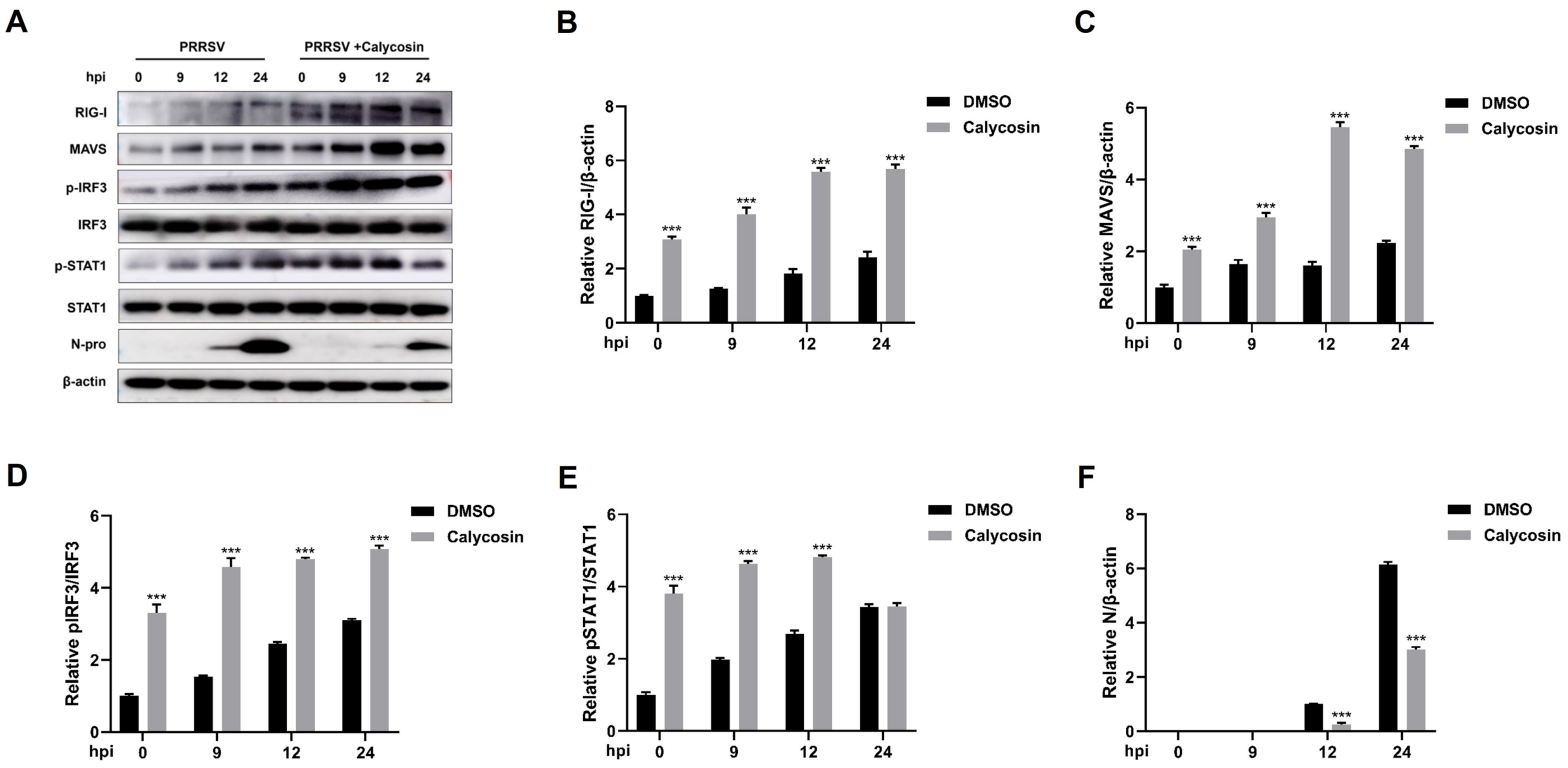
Calycosin promotes the activation of RIG-I/IRF3 signaling pathway. Marc-145 cells were mock treated or treated with calycosin (40 μg/mL) and then infected with PRRSV (MOI = 0.1) for different time periods. **(A)** The RIG-I, MAVS, pIRF3, IRF3, pSTAT1, STAT1, and PRRSV N protein were detected by western blotting. **(B–F)** The relative expression levels of RIG-I, MAVS, pIRF3, pSTAT1, and N protein were quantified using Image J software. The asterisks in the figures indicate significant differences (*p < 0.05, **p < 0.01, ***p < 0.001).

**Figure 10:**
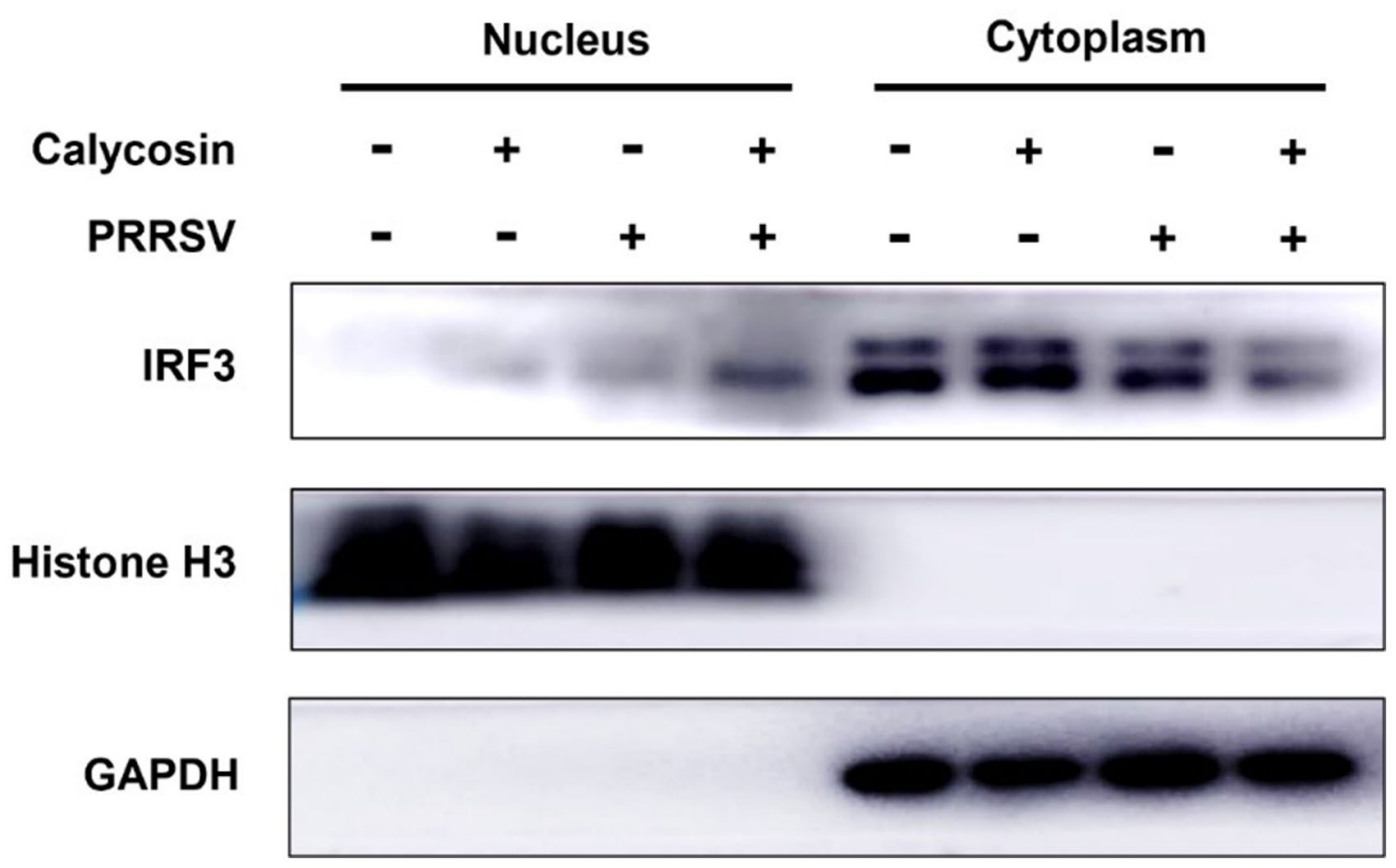
Calycosin treatment significantly promotes IRF3 nuclear translocation during PRRSV infection. Marc-145 cells were treated with calycosin (40 μg/mL) in the absence or presence of PRRSV (MOI = 0.1). After 12 h, the cells were collected, and the levels of IRF3 in the cytoplasmic and nuclear lysates were analyzed by western blotting with GAPDH and histone H3 as cytoplasmic fraction and nuclear fraction markers, respectively.

Following that, we investigated the expression of RIG-I and MAVS, along with the phosphorylation and nuclear translocation of IRF3 during calycosin treatment in PRRSV-infected cells. The results showed that compared to PRRSV-infected cells without calycosin treatment, calycosin treatment led to a significant upregulation in the expression levels of RIG-I and MAVS. Moreover, it effectively induced the activation of pIRF3 and pSTAT1. At the same time, the expression levels of PRRSV N protein were markedly lower in cells treated with calycosin (Figure 9). Additionally, we detected the nuclear translocation of IRF3 upon PRRSV and calycosin treatment. We found that calycosin treatment significantly induced IRF3 nuclear translocation during PRRSV infection (Figure 10). These data indicated that calycosin positively regulates the RIG-I/IRF3 signaling pathway, which in turn, may be intimately linked to the suppression of PRRSV replication.

## Discussion

PRRSV has been prevalent for more than three decades and remains one of the most significant pathogens within the global swine industry, resulting in substantial economic losses (31, 32). Currently, the prevention and control of PRRS are severely hampered by the rapid evolution and high genetic diversity of PRRSV (11, 33). Therefore, the development of novel antiviral agents is urgently needed to prevent and control PRRSV. Natural compounds and their derivatives have been reported to be important sources for developing new antiviral drugs (34).

In this study, we explored the relationship between calycosin and PRRSV, and found that calycosin effectively inhibited the replication of PRRSV and positively regulated the RIG-I/IRF3 signaling pathway. We first explored the biological role of calycosin in PRRSV infection, and found that calycosin could efficiently inhibit PRRSV replication in a dose-dependent manner. Furthermore, calycosin could directly reduce virus infectivity. Notably, we observed that calycosin could block virus replication, assembly, and release but not the binding and entry stages. Moreover, we demonstrated that calycosin enhanced the activation of RIG-I/IRF3 signaling, which may be related to the antiviral activity of calycosin.

Calycosin, the major isoflavonoid in Radix Astragali Mongolici, has been documented to be involved in various biological functions, such as cancer, inflammation, autophagy and apoptosis (35–38). Although studies have shown calycosin has strong activity against coxsackievirus B3 (CVB3), human immunodeficiency virus (HIV) and Kaposi’s sarcoma-associated herpesvirus (KSHV) (22, 39, 40), the role of calycosin in antiviral immunity is poorly characterized. In this study, we discovered that calycosin exerts a significant inhibitory effect on PRRSV replication. And when calycosin was co-incubated with PRRSV, a marked reduction in PRRSV infection was observed. Moreover, the EC_50_ of calycosin was 10.58 μg/mL. However, given that the maximum solubility concentration of calycosin is 100 μg/mL and that it has a propensity to precipitate when the concentration exceeds this threshold, calculating the CC_50_ and Selectivity Index (SI) of calycosin is impossible. Consequently, the inability to perform these calculations poses a limitation in the comprehensive evaluation of the therapeutic potential of calycosin. In addition, our research results merely offer preliminary evidence that calycosin can inhibit PRRSV replication. No comparative study has yet been conducted between calycosin and known inhibitors (e.g., Ribavirin or another well-characterized compound). A positive control in antiviral experiments not only validates the effectiveness of the experimental system but also serves as a reference standard for comparing the activity of new compounds. Although calycosin is not a novel compound and has demonstrated antiviral activity against other viruses, conducting in-depth research on the comparison of the inhibitory efficacy of calycosin and known inhibitors against PRRSV is important for evaluating the efficacy of calycosin and for future disease prevention and control.

Additionally, calycosin has the potential to impede the assembly, and release stages of the PRRSV life cycle. It has been reported that PRRSV GP2 and GP4 could interact with cellular receptor CD163, which is indispensable for PRRSV entry and release (41). Besides, GP5/M glycoprotein complex of PRRSV is also required for release (42). Therefore, calycosin might block virus release by disrupting the interactions between proteins or inhibiting the synthesis of related proteins. However, more research is needed to clarify this point.

Innate immunity is the first effective line of host defense against pathogenic microorganisms (15). RIG-I/MAVS signaling pathway is the key pathway to recognize and respond to RNA viruses (29, 43). When MAVS is engaged by RLRs, it recruits downstream signaling complexes that lead to the activation of IRFs and NF-κB (44). IRF3 activation is the hallmark of interferon pathway activation. In our study, we found that calycosin could enhance the expression of RIG-I and MAVS, and induce the phosphorylation and nuclear translocation of IRF3. Besides, NF-κB is also a crucial transcription factor for downstream signaling. And it has been reported that calycosin could modulate inflammation via suppressing TLR4/NF-κB pathway (45, 46). So, it is conceivable that calycosin may exert an inhibitory effect on PRRSV replication by regulating the NF-κB-mediated inflammatory response, which may provide valuable insights into the potential antiviral mechanism of calycosin. However, further studies are required.

In summary, our findings provide novel insights into how calycosin inhibits PRRSV infection. Here, we demonstrate that calycosin can activate the RIG-I/IRF3 signaling pathway and has the capacity to suppress PRRSV infection via inhibiting virus replication, assembly, and release, which highlights the potential of calycosin as a therapeutic agent for inhibiting PRRSV replication and provides new insights into antiviral strategies. However, further in-depth research is needed to determine whether calycosin can also inhibit other PRRSV strains or viruses. And future research should focus on the assessment of the antiviral activity of calycosin as well as its safety and efficacy *in vivo*, so that it can be used as an effective antiviral drug and widely applied in the prevention and treatment of animal diseases.

## Data Availability

The original contributions presented in the study are included in the article/supplementary material, further inquiries can be directed to the corresponding authors.
